# Harvesting *Bertholletia excelsa* Bonpl. in a western Amazon rural community: local ecological knowledge and meaning to “nut-crackers”

**DOI:** 10.1186/s13002-023-00635-y

**Published:** 2023-12-15

**Authors:** Arlene Oliveira Souza, Alessandra Rufino Santos, Sergio de Faria Lopes, Tathyna Rodrigues Soares

**Affiliations:** 1 Federal University of Roraima – UFRR, Av. Capitão Ene Garcêz, 2413, Bairro Aeroporto, Campus Do Paricarana, Boa Vista, Roraima CEP 69304-000 Brazil; 2 Postgraduate Program at the of the State University of Paraíba – UEPB, R. Baraúnas, 351 - Universitário, Campina Grande, PB 58429-500, Campina Grande, Brazil; 3 Escola Municipal, 12 de Outubro, Vicinal 09, Km 11, Caroebe, Brazil

**Keywords:** Ethnoecology, Amazonian chestnut, Traditional populations, Environmental changes, Local knowledge

## Abstract

**Introduction:**

The collection of Bertholletia excelsa Bonpl. (castanha-da-Amazônia; Brazil nuts) seeds make up part of the everyday activities of the traditional populations that have inhabited all of the Amazon basin since remote times. Nonetheless, knowledge about these harvesting activities in native forest areas has not been well documented. The present study was designed to better understand the significance of this harvesting activity as well as the traditional ecological knowledge of the harvesters.

**Methods:**

Utilizamos entrevistas semiestruturadas para a coleta de dados com extrativistas de castanha, moradores de Caroebe, Roraima (*n* = 18) durante o período de março de 2021 a março de 2022. The data analysis was based on the frequency of responses to socio-economic questions and their knowledge about that plant species as well as why those interviewees chose that line of work. We also adopted the Spearman and Mann–Whitney non-parametric tests to correlate variables identified in the study, and selected sections of their depositions to highlight the traditional knowledge of the interviewees and their experiences as harvesters.

**Results:**

Constatamos que as razões para a escolha do trabalho com o extrativismo para todos os entrevistados é a necessidade de renda complementar, tradição familiar (55%), the sense of well-being provided by contact with the natural environment (25%), and a favorable disposition toward that type of work (11%). Harvesting involves collective work, and many of the interviewees had engaged in those efforts to help their families since their childhood or adolescence. The older harvesters cited more animal species that consumed the Brazil nuts (*ρ* = 0.60; *p* = 0.009) and perceived more and greater changes in the environment that were prejudicial to the Brazil nut trees (*U* = 9.50; *p* = 0.022). The interviewees who reported lower incomes cited more significant cultural changes and more suggestions concerning conservation activities. According to their statements, deforestation, and the burning and illegal cutting of native trees, including Brazil nut trees, have contributed to environmental change in the region and raised significant concerns about the future of harvesting activities.

**Conclusion:**

The activities of the “nut-crackers” represent to them more than just a simple source of income, as harvesting provides them with a connection to nature that promotes their well-being and cultural heritage. The nut harvesters have gained specific knowledge concerning both environmental and cultural changes. Those changes have mainly come about through the expansion of agricultural activities and the felling of native forests—which are the main threats to the future of Brazil nut extraction. Attributing value to the folk knowledge of those harvesters would strengthen the local economy, promote forest conservation, and help to better understand the impacts of anthropic activities on the forest and the harvesting of natural products.

## Introduction

Food plants have been present in the daily lives of Amazonian populations since ancient times [1]. Archaeologists have found microscopic traces of *Bertholletia excelsa* Bonpl. (Brazil nut) in the charcoal of fire pits dating from the early and middle Holocene, approximately 10,000 years ago [2], representing proof of its traditional use as a food source. Official incentives and changes in the economic cycles of forest products (such as rubber extraction from *Hevea brasiliensis*), have promoted Brazil nut harvesting as an alternative source of income for many Amazonian communities [3–5].

Nut harvesting in Roraima State, in the extreme northern region of Brazil, is an important source of both food and income for families living in rural areas. Harvesting activities are exclusively carried out in intact native forests in the municipalities of Rorainópolis, Caracaraí, São João da Baliza, Caroebe, and São Luíz do Anauá [4]. That harvesting represents a traditional indigenous practice of the Wai Wai and Yanomami people, although it now also involves migrant farmers from different regions of Brazil [4–6]. The harvesting of Brazil nuts also occurs in forest areas in other South American countries such as Bolivia, which have vast areas of Amazon Forest cover. The low-income families there depend on those forests to an even greater extent than Brazilian families in the southwestern Amazon who have cattle raising as their principal economic activity [3].

The daily activities of the nut-crackers have provided them with a vast knowledge concerning biological and ecological aspects of *B. excels,* and about environmental disturbances that can affect nut production [7]. Through their nut collecting, for example, the indigenous Kayapó people contribute to the maintenance of their culture and reduce the attractiveness of predatory income sources such as logging and mining—resulting in a positive impact on the recruitment and dispersal of *B. excelsa* trees [8]. Likewise, harvesting nuts lends forest dwellers economic autonomy and guarantees the occupation of the quilombola territory in Oriximiná/PA. In that community, Brazil nut groves are given the name “Pontas de Castanha”, and their ecological knowledge is manifest through their careful management. The quilombola concept of “understanding” the Brazil nut refers to having detailed knowledge of the tree itself and the regional ecosystem, which includes, for example, seed quality and the most appropriate places for hunting, fishing, and gathering other plant species [9]. Therefore, attributing value to Brazil nut harvesting can promote conservation, the maintenance of the local culture, and the generational transmission of traditional knowledge—contributing to improvements in human life and the permanence of forest populations [10]. Such ethnoecological issues, however, have been little addressed in studies concerning *B. excelsa*.

Studies based on ethnoecological approaches are designed to explore human interactions with the environment, local practices, local knowledge, cosmovision, and to recognize traditional peoples as profound experts concerning their environments through their coexistence and traditional use of biodiversity. For this reason, their practices and knowledge must be recorded and valued [11] to better understand biodiversity and the effects of anthropic actions on species and natural ecosystems from a conservationist perspective [12–17]. Extending this discussion, [18] argues that human actions in the environments that surround them are supported by meanings based on social interactions and evolve over time. Symbolic interactionism is a method of analyzing human behavior, and this theoretical framework makes it possible to observe how humans act in relation to the universe, how the meanings of social interactions are internalized, and how those meanings are interpreted.

Here, our interests turn to an analysis based on an ethnoecological approach that is designed to better understand the multiple factors that shape the appropriation of biodiversity resources [19]. Its assumption is the appreciation of the cultural diversity manifested within each society as reflected in the phenomenon investigated [20]. In other words, an ethnoecological approach considers that each social group, in its geographic space, acts according to its unique vision of the world, thus composing the essential cultural, cognitive and practical aspects of the lives of its members [20–22]. We also seek support in the assumptions of Symbolic Interactionism to better understand the meanings attributed to extractive activities. The focus of this theory, according to [23], p. 130] is that they represent “the processes of interaction, that is, social action characterized by immediately reciprocal orientations”, with social relations being seen as open and subordinated to continuous recognition by the members of the community. For [24], symbolic interactionism proposes: (1) observing processes, such as how, for example, groups and societies arise, considering that nothing is fixed and static; (2) Investigating the meanings, symbols, and languages that engender social life; (3) Investigating interactions and interconnections, as they represent the best view that one can have of the individual, who is always interacting.

Three questions therefore guided our study: What are the motivations that give meaning to being a “nut-cracker”? Is there a relationship between socioeconomic variables and local ecological knowledge? These questions are linked to understanding the meaning of harvesting activities and the knowledge “nut-crackers” have acquired concerning *B. excelsa*, in the municipality of Caroebe in southern Roraima State, Brazil.

We accepted as a hypothesis that the motivation for becoming a Brazil nut harvester is the necessity of obtaining additional income and that socio-economic variables such as age, income, schooling, and time of working as a harvester, are related to local ecological knowledge.

## Materials and methods

### Study area

**Fig. 1:**
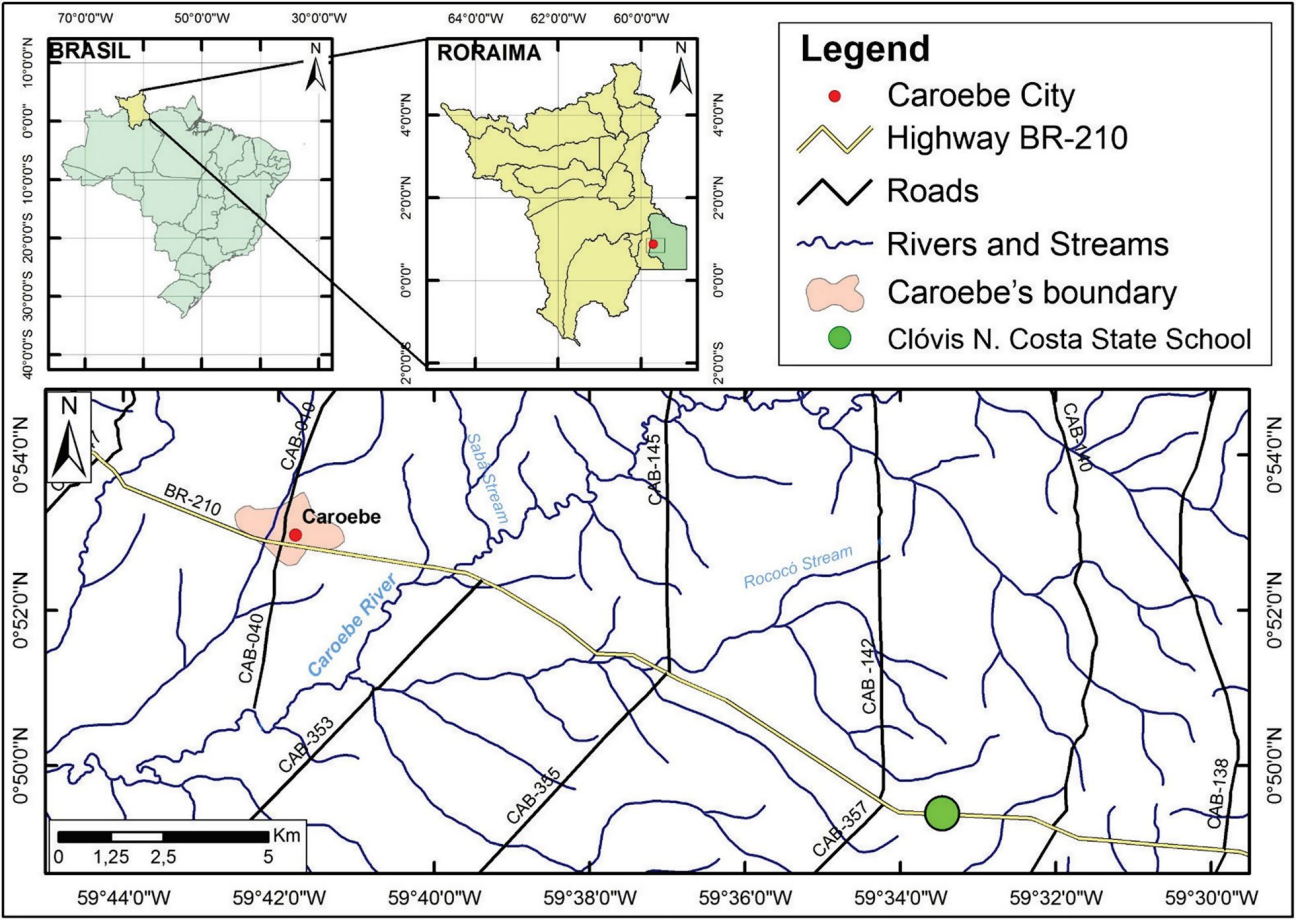
Location of the municipality of Caroebe in Roraima State, Brazil. Author: Boto, 2023

The municipality of Caroebe is located in the southern region of Roraima State, 354 km from the state capital of Boa Vista, Brazil. Caroebe’s, together with the municipalities of Rorainópolis, São Luiz do Anauá, and São João da Baliza, constitute the southeastern micro-region of the state [25], as illustrated in Fig. 1.

The predominant vegetation in southeastern Roraima is Dense Submontane Ombrophylous Forest [26].^1^ The regional climate there is characterized by a defined dry season from December to March, corresponding to type Am of the Köppen classification system.

The average annual rainfall in the region ranges from 1700 to 2000 mm, with the maximum (40% of all rainfall) occurring from May through July [27].

The economy of Caroebe is based on agricultural activities, with emphasis on food production (mainly bananas and cocoa) and cattle raising [28], in addition to the exploitation of non-timber forest products such as nuts, vines, and oils. The local population is principally composed of non-indigenous people and migrants from different parts of Brazil [29].

### Data collection

The participants in this study were nut collectors of both genders, over 18 years old, who were residents of the Caroebe municipality; they were non-indigenous and all were Brazilians. The snowball technique was used to select the interviewees [30, 31], following the initial interview of an experienced Brazil nut harvester who then indicated other harvesters, and so on successively until we noted a saturation of their responses. To avoid sample biasing, we included only interviewees with harvesting experience and who resided for the longest periods in the municipality. Data collection was carried out through semi-structured interviews [32] during visits to the nut harvesters’ homes in the local area.^2^

We used a standardized questionnaire covering socio-economic aspects and the gatherers’ personal knowledge about the plant species and environmental and cultural changes related to nut harvesting in Caroebe (Table 1). Our ecological knowledge proxies, as highlighted in the aforementioned, considered the frequencies (quantity) of the responses of the individuals concerning changes in the production of Brazil nuts, perceived environmental alterations, animals that feed on Brazil nuts, and perceived risks of the extinction of those trees.

**Table 1:** Semi-structured Research Questionnaire

Code	Description
ID	Interviewee name
GNDER	Gender
ORIGIN	Origin
AGE	Age
HOME	Local home
TIME-HOM	Time home (years)
EDU	Schooling level
PROFESS	Profession?
TIME-ACT	Time harvesting? (years)
OTHER	Other economic activities ?
ACTIV	What activity?
QTD-OTH-ACT	Number of other activity besides harvesting
INCOME	Family income (minimum wage)
INCOME_ACT	Income only from harvesting (% INCOME)
INCOME-OTHER	Besides harvesting income, is there another?
INCOME-OTHER	Origin of other income?
INCOME_ORI_AMT	Amount of income other than harvesting?
	From an economic point of view, are you satisfied with harvesting?
SATISFIED	
SIGNIFICANCE	What does harvesting signify to you?
REASONS	Why did you chose harvesting?
REASONS_QTD	Number of reasons
IMPORTANT	Why is the forest important to you?
ALONE	Do you harvest alone?
ALONE_WHO	Who accompanies you?
PERIOD	Period of harvesting?
CHANGE	Have observed changes in fruit production?
CHANES-WHAT	What changes have been observed?
CHANGES_QTD	Quantity of changes observed
CHANGES_ENV	Noticed environmental changes that could hurt harvesting?
CHANGES_ENV_WHT	What changes?
CHANGES_ENV_QTD	Quantity of environmental changes observed
CONSERVATION	What contributes to the conservation of Brazil nut trees?
CONSERVATION_QTD	Quantity of contributions observed
ANIMALS	Which animals eat Brazil nuts?
ANIMAIS_QTD	Quantity of animals observed
DIFFICULTY	What difficulties encountered while harvesting?
DIFIFICULTY_WHT	What are those difficulties?
RISKS	Is there a risk of not having nuts in the future?
RISKS_WHT	What are those risks?
RISKS_QTD	Quantity of perceived risks
LEARNED	With whom did you learn about harvesting?
FAMILY	Besides you, does anyone else in your family harvest?
FAMILY_WHO	Who in your family harvests?
CULTURE	In your opinion, have there been cultural changes in collecting?
CULTURE_WHT	What were those cultural changes?
PRODUTIVE	
PRODUTIVE_QTD	35–––––––––––––––––––––––478
USES	Uses

The questions shaded in gray were analyzed quantitatively, as they deal with structural questions. The other questions (in white) were analyzed qualitatively, as they deal with non-structural questions. The questions with the _QTD suffix were created by the researchers and concern *proxy* variables of ecological knowledge, and refer to the quantity of items that the individual mentioned in that particular question. For example, in the variable ANIMALS, if the individual identified an arara, parrot, monkey, deer, and a pig eating Brazil nut fruits, the value (score) of the variable ANIMALS_QTD would be 5

### Data analysis

The interviews were recorded, transcribed, and subsequently systematized; the data frequency is presented in percentages, based on the total number of respondents. The significance of being a Brazil nut harvester is sustained by the symbolic interactions with nature and the socioeconomic perspectives of that work. For a better comprehension of that symbolism, we undertook descriptive and bivariate analyses of the data, correlating the variable measuring the quantity of reasons mentioned for choosing to work as a Brazil not harvester with the variables income, schooling, and time dedicated to that activity. The variable Traditional Ecological Knowledge concerning the species and cultural changes (the dynamics of collective family work) was correlated with income, schooling, and age.

For the bivariate analysis, non-parametric tests were adopted for independent samples (mainly because of the sample size) and occasionally statistical tests between the scalar/ordinal and nominal variables. We therefore chose crossing the scalar, ordinal, and counting variables, based on Spearman's correlation coefficient (*ρ*), and applying the Mann–Whitney (*U*) test between nominal variables with only two classes and scalar/nominal variables [33]. Quantitative data were processed and analyzed using SPSS software, version 23.0 [34]. To more completely present the traditional knowledge and experiences of the interviewees, we selected excerpts from their responses. The anonymity of the interviewees was maintained for ethical reasons.

### 2.4 Research legalization

As the study involved access to traditional knowledge, approval to carry out the research was obtained from the Ethics Committee for Research with Human Beings at the Federal University of Roraima (UFRR), (Opinion 4,642,807), in accordance with legal and institutional requirements. The Free and Informed Consent Form (TCLE) was signed by all interviewees.

## Results

### 3.1 Research participant profiles

Most of the Brazil nut harvesters interviewed in this study were migrants from other Brazilian states (83% *n* = 18), especially the states of Maranhão (44.4%) and Pará (16.7%). Their ages ranged from 18 to 71, with males predominating; only one woman participated in the study. She reported that more women were working when she started harvesting Brazil nuts in the 1980s, but due to the difficulties inherent in that work and the necessity of conciliating harvesting with domestic activities (or health considerations), many left the workforce. All of the interviewees were married and lived in the municipality of Caroebe (Table 2).

**Table 2:** Socio-demographic profile of the nut-harvesters interviewed in the municipality of Caroebe in southern Roraima State, Brazil

Gender	Origin (State)	Age	Time living in Caroebe (years)	Education	Time working (years)
Male	Rorama	18	18	High school complete	8
Male	Pará	22	7	Fundamental incomplete	5
Male	Maranhão	23	13	Superior incomplete	8
Male	Roraima	24	24	Illiterate	3
Male	Roraima	26	16	Superior complete	12
Male	Roraima	29	22	High school complete	10
Male	Maranhão	31	25	Fundamental incomplete	7
Male	Rondonia	31	9	Funda mental incomplete	6
Male	Maranhão	36	13	High school incomplete	13
Male	Maranhão	36	8	Fundamento incomplete	8
Male	Minas Gerais	42	8	Superior complete	2
Male	Mato Grosso	43	38	High school complete	10
Male	Maranhão	58	20	Fundamental incomplete	14
Male	Pará	59	38	Illiterate	38
Male	Pará	59	12	Fundamental incomplete	12
Female	Maranhão	61	33	Fundamental incomplete	10
Maculino	Maranhão	70	34	Fundamental incomplete	10
Male	Maranhão	71	35	Fundamental incomplete	22

The education levels of the interviewees varied; most had not completed elementary school (61.1%), and a high school education was achieved by only a few (17%); one had completed a teaching degree (5.6%) in Rural Education, but currently performs agricultural activities and Brazil nut harvesting, commercialization, and as a middleman.

Many of the interviewees (66.7%) work as farmers, mainly in the cultivation of bananas; other occupations include: cattle and pig raising, handicraft production, making brooms, driving, teaching, selling clothes or Brazil nut by-products, and topographic services. Despite income from these activities and emergency aid and/or welfare payments (33%) from the federal government, the average family income is less than two minimum wages (US$ 445.03 ± US$ 340,87). When asked about their income only from Brazil nut harvesting the interviewees were unable to provide a precise answer due to wide price fluctuations.^3^

### The significance of harvesting activities to the “nut-crackers”

The analyses of the interviews with persons who identified themselves as “nut-crackers” revealed that the decision to harvest forest resources was associated, above all, with the perspective of financial gain. All of the interviewees emphasized the necessity of supplementing their incomes as a reason for becoming a Brazil nut harvester. The interviewees also mentioned family traditions (55%) because, when they were children, their parents took them (and other family members) to help gather the nuts; this still occurs today. Other interviewees expressed experiencing pleasure from being in contact with nature (25%) and liking their work (11.1%). These were the principal motivations to initiate and continue harvesting activities. The interviewees expressed pleasure from “remaining longer in the natural environment”, as expressed in the following quote from a Brazil nut harvester when referring to forest areas:

“I'd like to go crack Brazil nuts. Sometimes I break eight to ten sacks of nuts a day. I like being in the woods. I'd like to be in the rain all day. It was six o'clock in the morning with rain and rain. When I got home at five o'clock, it was still drizzling. Sometimes, I slept there” (QCMANP).

Satisfaction with harvesting activities was demonstrated by a significant percentage of interviewees (77.8%) who noted that Brazil nuts were naturally offered by the forest. “It is not necessary to plant”, and their living conditions were greatly improved through the supplementary income generated from the sale of those nuts. The Brazil nuts were therefore praised by the interviewees. A few, however, expressed dissatisfaction with the work (5.6%), citing the dangers and difficulties inherent to the work. Others were unable to answer the question (16.7%):

“First of all, I thank God for everything that gave me this direction. Today, I'm here in this place. And then Brazil nuts, because they opened the door for me to have what I have today” (QCMFCS).

“I practically built my own house in Caroebe with cash from Brazil nuts. I've fixed up a lot of little things for myself with that Brazil nut money. So it pays off financially. It also compensates for the service, the work that is done going into the forest and breaking the fruit cases (ouriços)” (QCMAMF).

It makes a lot of money, but it takes a lot of work too.” (QCMJFSS).

Nut-crackers dedicate their efforts to harvesting Brazil nuts for at least half of the year, a period during which the organization of their daily activities is guided by the idea of ​​the usefulness of the nuts and their seasonal production. In this sense, the monetary value of the harvested nuts becomes central, although their value varies annually according to supply and demand. In 2022, they sold for US$48.82/sack, while in the previous year, they sold for US$ 62.50/sack.

**Fig. 2:**
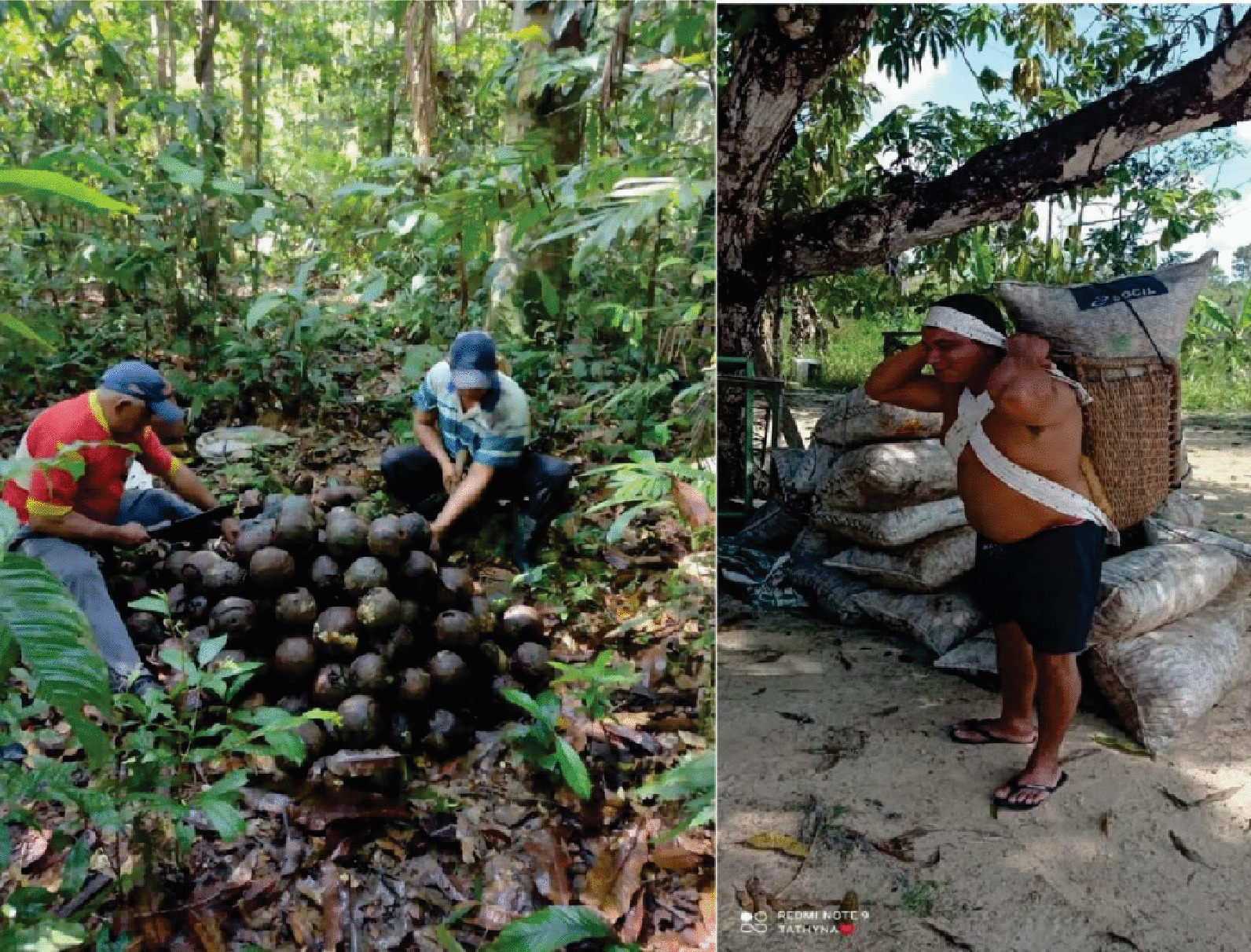
Images (**a**,** b**) of the extractive activity of Brazil nut (*B. excelsa*) in the municipality of Caroebe, state of Roraima, Brazil. Source: Soares, 2021

Harvesting requires physical exertion and exposure to natural dangers, with the risk of accidents and attacks by wild animals. The harvesters enter areas of native forest vegetation to collect the seed capsules, which are piled up and then broken to remove the seeds. The seeds are subsequently bagged and transported to tractors and then to the points of sale. All of the interviewees highlighted the efforts of carrying bags of Brazil nuts on their backs over long distances through the forest as one of the difficulties of their work. Some of them use a *remanchin*, the local name given to three-sided carrying baskets made from the vines of forest species and produced locally by one of the interviewees (Fig. 2).

Regarding other uses of Brazil nuts, the interviewees noted that their extracted oil is used to prepare foods such as rice, beans, and meat. Milk derived from the crushed seeds is consumed together with bush meat. The nuts themselves are eaten *in natura,* and flour and other food items are produced from it, such as *cocadas* and homemade sweets, which are also sold locally. The heavy fruit case (*ouriço*) is used to make organic fertilizer and crude bowls (*cuias*). The central part of the fruit (*umbigo*) is used to treat diabetes.

According to the statistical analysis, there was no correlation between the variables family income and quantitative reasons for being a Brazil nut harvester (*ρ* = 0.15; *p*-value = 0.59). However, there was a difference between those who had spent more time as a harvester (more than 5 years) and those who declared that they had other sources of income when comparing the reasons they expressed for being a nut harvester (*ρ* = 007; *ρ* = 001), indicating that Brazil nut harvesting is a complementary alternative to family income. Likewise, there was a relationship between the variable levels of education and the number of reasons for being an extractivist (*ρ* = 0.55; *p*-value = 0.02). This is probably due to the variation in education levels, as the sample ranged from illiterate individuals to those with higher education. However, most respondents had low levels of education, and the work under discussion does not require schooling.

## Local ecological knowledge of caroebe nut harvesters

Nut harvesting is a traditional practice and is generally undertaken collectively. Fully 83% of the nut harvesters stated that they go into forest areas to gather Brazil nuts while accompanied by family and/or friends.^4^ Some of them reported that they had started working during childhood or adolescence and that they learned about that work by helping their parents.^5^ Additionally, the interviewees often had extensive experience harvesting Brazil nuts and significant times of residence in the municipality (20.72 ± 10.94 years), with 39% of them starting harvesting as soon as they arrived in Caroebe in the 1980s.^6^

Forest areas in the public domain were better preserved at that time, and Caroebe was still a district of São João da Baliza.

When interviewed, most nut-crackers (55.5%) had been harvesting Brazil nuts for at least a decade. There was a positive correlation between age and working time (*ρ* = 0.60; *p*-value = 0.008). A longer working relationship with a plant species favors the accumulation of knowledge about it. The older interviewees cited more animals that fed on Brazil nuts (*ρ* = 0.60; *p*-value = 0.009); there was no correlation, however, with education (*ρ* = −0.137; *p*-value = 0.60). Various animal ethnospecies were mentioned in detail during the interviews, demonstrating extensive ecological knowledge: macaws, monkeys, paca, wild pigs, birds (black chick, woodpecker), deer, capybara, squirrels, tapir, and agouti. The latter is recognized as the principal disperser of *B. excelsa*:

“[...] several wild animals eat Brazil nuts. The macaw drops them to the ground. The monkey and other animals eat them, both deer and pigs. The deer eat the flowers. The macaw eats them when they are still green. When they are green, they are soft. Then, the macaw drops them. They are soft and then it gnaws and eats them” (QCMMSSS).

“[...] when the nut is small, the macaw and parrot eat a lot” (QCMTSS)

“[...] the monkey, when we walk through the forest, we see that it throws nuts on the ground. He goes up there and hits them "toc, toc". Then, when he throws the nut, we go there to look. Then, there's a hole there in the fruit, all he has to do is put in his finger. Sometimes, they find a little gap for him to take the nut” (QCMANP).

Due to the importance of the nuts to the local community, the future of harvesting was a matter of concern for the interviewees. According to them, deforestation, burning, and the illegal cutting of native trees, including Brazil nut trees, were contributing to local environmental alterations. Although the law expressly prohibits cutting down *B. excelsa*, they reported that Brazil nut trees were being felled in the municipality.

The older nut harvesters (*ρ* = 0.57; *p*-value = 0.013) also had other sources of income (*U* = 9.50; *p*-value = 0.022), and mentioned more changes in the environment. According to them, the changes were caused by human actions that impacted Brazil nut productivity, with the main threats to *B. excelsa* being deforestation which results in decreased rainfall caused by increasing local temperatures and smoke from wildfires that delays the flowering of the Brazil nut trees. “*Where there is a lot of clearing of the forest and smoke, the chestnut trees take a long time to load*”, as observed in the following narratives:

“Fire damages nuts a lot. If she feels the heat, she usually dies. And also, the burning damages the nuts a lot because of the bee. The bee that pollinates where that fire passes. That smoke kills all the bees. There is no way to germinate” (QCMFCS).

“Caroebe is a region that stands out among other states, a region that is very rich in Brazil nuts. And lately, it's not raining. This affects production because of deforestation, right? Extraction of the wood itself and the burning of nature. In this case, due to deforestation for cattle raising. So, they end up doing this deforestation and thinning out nature” (QCMWNR).

“The forest is cut down by farmers and others who are acquiring land here and the fires interfere with flowering, the Brazil nut tree flowered right there. For some time now, because of the smoke, it blooms, but the flowers fall, they do not hold” (QCMANP).

Cultural changes and contributions to the conservation of Brazil nut trees were also mentioned by the nut harvesters, mainly by having lower income levels (U = 6.50; *p*-value = 0.005).^7^ Among the changes noted was that memories were shared that the families stayed longer in the forest in the past because “*they had no one to leave the children with*”; today, this is no longer the case. Only a few relatives or friends participate in harvesting, and some even prefer to go alone (11%). Traveling to nut collection sites also changed, as before people traveled along footpaths (*picadas*) through the forest to access Brazil nut stands. Now there are openings within forest areas giving better access to stands. Currently, a good part of the journey to collect Brazil nuts is made by motorcycle, and they are left near the edge of the forest.

The interviewees also mentioned changes in conservationist actions, especially: “reforestation, preservation of forest areas, stopping burning and the cutting down of forests; respect for environmental legislation; the transfer of knowledge to younger people; and the protection of pollinators to avoid reductions of Brazil nut production”. No relationship was observed, however, between the variables amount of conservation actions and schooling (*ρ* = 0.03; *p*-value = 0.89), or with the time participating in harvesting activities (*ρ* = 0.04; *p*-value = 0.88).

“Each landowner must maintain a good buffer zone for Brazil nut trees and the vegetation around it when clearing and planting (QCMRTS)”

“Forest preservation, respect for legislation, and transfer of knowledge” (QCM DSS)

“Do not cut down the forest, and replanting with Brazil nut trees” (QCMANP).

## Discussion

### The meaning of harvesting activity

Harvesting activities have dimensions deeply connected with the possibility of financial gain, family traditions, and a sense of well-being which is favored by direct contact with the natural environment and the pleasure of working as a “nut-cracker”. Other social and cultural elements are symbolically and concomitantly constructed during the time dedicated to the activity and the experiences gained, corroborating the idea that human actions are based on the meanings that the world offers them [18]. Therefore, the decision to appropriate forest resources attributes economic, cultural, and sentimental meaning to that activity. This feeling of well-being by the interviewees justifies going into the forest to search for Brazil nuts and face the risks inherent to the job.

The low level of education of most workers adds to this feeling and has influenced the choice for this activity, as Brazil nut harvesting does not require any formal schooling. People with higher education levels in rural and urban–rural areas usually occupy jobs unrelated to outdoor work [36]. Additionally, the other uses of Brazil nut resources mentioned by the interviewees, such as food, traditional medicine, the production of organic fertilizer, and the commercialization of nut by-products must be considered because these secondary forest resources—leaves, fruits, bark, roots, oils, resins, and seeds—land meaning to rural life due to their utility and to their contributions to the livelihoods of human populations [37].

The value attributed to local nut extraction by the interviewees can also be seen in the satisfactory assessment of those forest resources by almost all of the interviewees. There were complaints, however, about annual price variations of Brazil nuts, as well as mentions of work difficulties due to the physical efforts required and the vigilance necessary in forest environments to avoid accidents involving wild animals and being hit by falling fruits. These circumstances explained why harvesters almost always were accompanied while working in forest areas.

The meanings of harvesting activity can therefore be transformed according to the social context experienced and interpreted by the nut-crackers, giving meaning to their attitudes [18]. The interactions stimulated by the ability to interpret and perceive the symbols surrounding the interviewees give harvesting activities meaning as a source of complementary income, the enjoyment of the goods offered by nature, and personal satisfaction. This activity, however, also requires hard and dangerous work. The actions motivated by the interactions of the Brazil nut breakers is called symbolic interactionism, and through them, the meaning of the information is constructed, or reconstructed, according to the social and cultural contexts of each individual [18].

From this perspective, and through the understanding and speculations of the research interlocutors, it can be understood that symbolic interactionism attributes symbolic meaning to Brazil nut harvesting in Caroebe. These same meanings, in turn, encompass the otherness and the identities constructed by the “nut breakers”, allowing evidence of local ecological knowledge to appear.

### Local ecological knowledge of the “nut-breakers”

The results of the statistical analyses confirmed relationships among some of the analyzed variables. Respondents with low incomes and low education levels, who had dedicated more years to collecting nuts, demonstrated greater in-depth ecological knowledge of *B. excelsa*. It would be expected that spending more time interacting with the natural environment would contribute positively to the acquisition of knowledge [36]. The harvesters having longer experience at that work indicated with precision the animals that fed on Brazil nuts, and they could detail how the seeds were consumed. They recognized, for example, the role of the agouti as a seed disperser. These data confirm the results of the study by [38] showing that the broad knowledge of native forest species demonstrated by family farmers is related to their life experiences. These authors also emphasized that abandoning the use of forest species leads to the loss of that knowledge.

Likewise, the elderly and more experienced nut-crackers, as well as those with other sources of income, recognized more human actions causing environmental changes harmful to the productivity of Brazil nut trees. It is important to note that their alternative incomes are derived from agricultural activities, government aid, and other occupations such as handicraft production and broom-making that use raw materials from the forest. In other words, these alternative income sources demand other types of management skills and the appropriation of native, non-forest resources and, consequently, demand additional environmental knowledge.

The felling of trees, including Brazil nut trees, and the burning of forest areas for logging and agricultural activities were cited as the main perceived threats to forest ecosystems. Those activities were identified as causing negative impacts on harvesting through the decreased productivity of Brazil nut trees. It should be noted that that view is supported by records of significant losses of native vegetation in Roraima (with 203 km^2^ being deforested from 2019 to 2021—a more than 100% increase in lost forest area as compared to the period between 2016 and 2018) [39].

Cultural changes were recognized and better explained by interviewees in the lower income categories, who put forth suggestions for conservation actions to protect Brazil nut trees (and, consequently, their harvesting). Their responses indicated that harvesting is a traditional activity, although it has undergone cultural changes. The interviewees reported that all family members would gather in the forest in the past and stay for weeks at a time harvesting nuts. Thus, some workers began harvesting Brazil nuts to help their parents, for there was no one else to leave them with. Other harvesters were encouraged by family members, cousins, in-laws, in-laws and/or friends, to venture into forest areas alone. This information denotes the tradition of extractivism and the transmission of knowledge between generations, reinforcing group identity [40]. It also became clear that knowledge was also acquired and transmitted through individual observation and experimentation [41].

Within this context, [42] was justified in stating that where there is a predominance of a traditional culture, the workforce becomes latent and of common use. This is evident in Caroebe, where the transmission of traditional knowledge based on nut extraction became efficient because a number of current workers in the nut harvesting and logistics processes had contact with this environment while still in their infancy.

Making a counterpoint to cultural changes and contributions to the conservation of chestnut trees, [43] makes it clear that the market, in which traditional populations operate today, is not the same as in the past. This is a reflection of how traditional knowledge has changed, for just as the market has changed, nut collection itself is not as it was before.

In this sense [20–44], noted that the coexistence of traditional peoples, such as the Brazil nut-crackers, living close to or in anthropic areas in the Amazonian, favors the production of knowledge and an understanding of socio-environmental transformations in these ecosystems. Socio-ecological systems, however, can be compromised by rapid environmental changes, and there may be losses of local knowledge due to the inability to adapt to these changes [45].

It is important to note that the interface between Ethno-ecology and Symbolic Interactionism proposed in this study can be demonstrated through the traditional knowledge of the “nut-crackers”. During the interviews, which revealed important elements of the culture of the social group, and the analyses of their regular everyday interactions and practices, it was possible to understand the tenacity of the continuity of this activity through the years.

This study verified that a dialogue between Symbolic Interactionism and Ethno-ecology can provide important clues about the relationships between the nut-crackers and the society within the municipality of Caroebe/RR, making it clear that its projects and trajectories do not depend solely on the virtues of desires, but are often conditioned by the social context in which they are immersed.

In summary, both approaches allow the view that the individual trajectories of the “nut-crackers” are the results of the relationship between personal projects and the social world, proportioning a work relationship in close contact with the environment in which they are inserted.

## Conclusions

Harvesting Brazil nuts has economic, cultural, and sentimental meaning for “nut-crackers” in the municipality of Caroebe, as the nut is offered free of charge by nature. It is possible to obtain additional income from those nuts, as well as to enjoy a feeling of well-being through rewarding work and experiencing the natural environment.

The interviewees acquired knowledge through their harvesting practices and contact with the *B. excelsa*, and have passed it on to other generations. The oldest nut-crackers, having spent more time in a working relationship with the species, demonstrated significant knowledge about ecological aspects, and had observed changes in the forest ecosystems due to human actions harmful to Brazil nut productivity. That knowledge will be important for future planning of conservation actions, not only for *B. excelsa*, but also for natural forest ecosystems that are essential for maintaining the ecological equilibrium in the Amazon region.

From the point of view of the theory of symbolic interactionism [18], it is clear that the knowledge of the “nut-crackers” concerning *B. excelsa* allows them to make interpretations that guarantee the execution of their daily activities and program their future activities. The results of this study support the importance of valuing local ecological knowledge and the participation of “nut-breakers” in conservation strategies for the harvesting of plant species and defending the sustainable use and conservation of natural resources and forest ecosystems.

Local ecological knowledge is found where traditional and indigenous populations live and work. In this case, the municipality of Caroebe can be considered a site that has generated one of the most efficient forms of orally transmitted historical records. As such, in this context, the narratives of the “nut-crackers” highlight a relevant culture and portray situations of heavy labor and suffering, but also great happiness.

## Data Availability

We declare that the data support the discoveries in the present study and are available from the corresponding author upon a reasonable request. Arlene Oliveira Souza, e-mail: arlene.oliveira@ufrr.br.

## Abbreviations

UFRR: Federal University of Roraima
PTDRS: Territorial Plan for Sustainable Rural Development
TCLE: Free and Informed Consent Form

## Footnotes

1 State Law n. 82/94 created the municipality of Caroebe, on November 4, 1994, separating it from the municipality of São João da Baliza. However, its official installation took place on January 1, 1997, with the inauguration of its first mayor, Mr. Antônio de Sousa Martins Filho, and the vice-mayor, Mr. Ilário José de Lima (PTDRS, 2010).

2 Caroebe's land structure is based on the PAD - Jatapu (Directed Settlement Project), sponsored by INCRA. There are also several neighboring settlements, sponsored by the state of Roraima, with lot sizes of up to 100 hectares predominating (PTDRS, 2010).

3 Individuals who purchase Brazil nuts locally and then sell them to buyers from other states and bordering countries (such as Guyana).

4 *Ouriços* is the common name given to Brazil nut fruits, which are woody and round [35].

5 Often used as a cup or food container.

6 The soft material found inside the Brazil nut fruit.

7 Federal law nº. 1.228, of 19 October 1994.
